# Radiation Exposure and Surgeon Safety During Minimally Invasive Spine Surgery in the Absence of Neuronavigation: A UK Single-Centre Retrospective Study

**DOI:** 10.7759/cureus.99209

**Published:** 2025-12-14

**Authors:** Azam A Baig, Evangelos N Anagnostou, Katlyn Green, Alexandru Budu

**Affiliations:** 1 Department of Neurosurgery, Queen Elizabeth Hospital Birmingham, Birmingham, GBR; 2 Neurosurgery, King's College Hospital London, London, GBR

**Keywords:** minimally invasive spine surgery, neuro-navigation, radiation dose reduction, robotic spine surgery, surgeon’s experience

## Abstract

Background: Minimally invasive spine (MIS) surgery has evolved significantly during the past decades; however, it remains dependent on fluoroscopic imaging in many centres throughout the world. Substantial radiation exposure to the patient and surgeon is inevitable in these procedures. The aim of this study is to assess whether MIS surgery without neuro-navigation (NN) leads to excessive radiation exposure.

Methods: We retrospectively analysed all spinal instrumented cases performed at a Level 1 Trauma Centre during a 12-month period. Number of X-rays and radiation generated were measured for both open and MIS cases and compared based on type of procedure and number of levels fused. Dose area product (DAP) dose registered at the end of the case by the X-ray machine. Body Mass Index (BMI) was also analysed to assess the impact it has on X-ray tissue penetration.

Results: In total, 141 instrumented thoracolumbar spinal cases were recorded, 57 MIS and 84 open, with a mean patient age of 55.8 and 57.5 years respectively. Mean BMI was 27.8 for the MIS group and 27.4 for the open group. For MIS cases, a mean of 192.3 X-rays were performed for each case, compared to 89.4 for open cases, while the dose calculated was 0.96 mGym2 for MIS, compared to 0.37 mGym2 for open.

Conclusion: MIS procedures without the use of NN carry a high risk of radiation exposure to the surgeon. Future research should focus on identifying barriers to the widespread adoption of NN.

## Introduction

Spinal surgery has evolved significantly over the past decades, with approximately 500,000 patients undergoing lumbar spine surgeries each year in the United States and over 50,000 patients in the NHS in the UK [1,2]. During this period, advancements in surgical techniques and medical technologies, including the increasing use of minimally invasive techniques and neuronavigation, aim to improve patient safety and outcomes [3].

Minimally invasive spine (MIS) surgery has become increasingly popular due to several demonstrated benefits, including smaller incisions, less soft tissue disruption, less postoperative pain, decreased blood loss, and faster recovery time [3-5]. However, one of the potential downsides of MIS surgery is the increased risk of radiation exposure to both the patient and the surgical team [6]. Fluoroscopy is commonly used in instrumented spinal procedures to aid in visualization and guide instrumentation placement, but it can also result in significant radiation exposure if proper precautions are not taken. Lead aprons and glasses are commonly used as an adjunct to protect surgical personnel from radiation exposure, but they don’t eliminate the risks [7]. The use of neuro-navigation (NN) technology in MIS procedures has been shown to significantly reduce radiation exposure to the surgical team and potentially the patient, while also improving surgical accuracy and outcomes [8]. Although neuronavigation has shown significant benefits, its adoption in MIS procedures remains limited, predominantly due to financial reasons but also due to surgical reluctance.

This study aims to explore the radiation burden in a large UK centre where NN is not yet employed, to investigate the amount of radiation exposure to both patient and surgical personnel during MIS surgery and open spinal instrumented cases under fluoroscopy, investigate whether there is a radiation exposure shortfall of MIS surgery in specific without the use of NN technology to improve safety for both patients and surgical teams.

## Materials and methods

Study design and population

A retrospective database review was conducted to analyze demographics and case characteristics for all instrumented spinal surgeries performed over a 12-month period. Anterior approaches to the cervical spine were excluded due to the differences in approach, technique and need for fluoroscopic guidance.

Study measures and statistical analysis methods

Mean radiation dose exposure was calculated for all instrumented spinal cases using the mobile intensifier report issued at the end of the procedure. Factors potentially affecting radiation dose, including surgical approach (open vs. MIS surgery), spinal region (cervical, thoracic, or lumbosacral), and patient characteristics (BMI, age), were analysed. Radiation dose measurements were obtained using total dose area product (DAP) to assess radiation risk, defined as the absorbed dose multiplied by the area irradiated (measured in mGy/m2 (Milligray per square meter)). Comparisons were made between groups with t-test or Whitney-Mann test when appropriate after evaluation of normality of distribution (Shapiro-Wilk test).

## Results

There were fewer MIS surgeries (n=57) in this cohort than open (n=84). Patients undergoing MIS surgery on average were two years younger than patients in the open group. The male-to-female ratio was 1.45:1 for the MIS surgery group and 0.74:1 in the open group. Mean BMI was calculated in both groups and showed almost identical averages of 27.8. Lumbosacral procedures were the most common (n=70, 49.6% of all surgeries), followed by thoracic (n=52, 36.9%) and cervico-thoracic (n=19, 13.5%) procedures (Table 1). 

**Table 1:** Characteristics and level of pathology for patients undergoing minimally invasive spine (MIS) and open surgery

	MIS	Open
Patient characteristics		
Age (years)	55.8 (±17.8)	57.5 (±15.7)
M/F	1.45:1	0.74:1
BMI	27.8 (±5.8)	27.4 (±5.8)
Level of pathology		
Cervical	1	18
Thoracic	22	30
Lumbosacral	34	36

**Figure 1:**
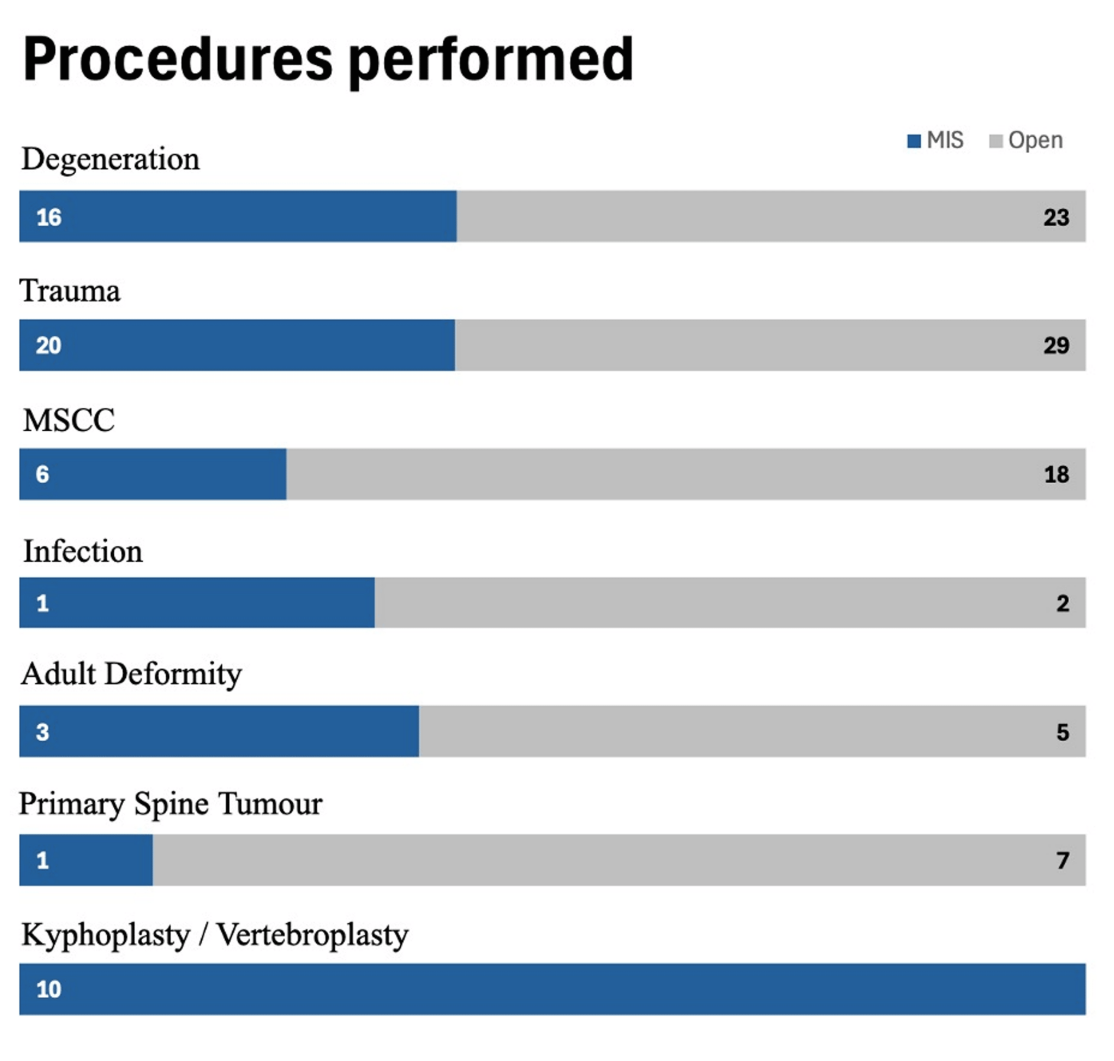
Number of minimally invasive spine (MIS) and open procedures performed as per indication MSCC: Metastatic Spinal Cord Compression

Surgeries for degenerative spine conditions, trauma, metastatic spinal cord compression (MSCC), infection, deformity, and spinal tumours were more often performed via the open approach. As expected, kyphoplasty/vertebroplasty procedures were performed with an MIS technique (100%) (Figure 1). 

**Figure 2:**
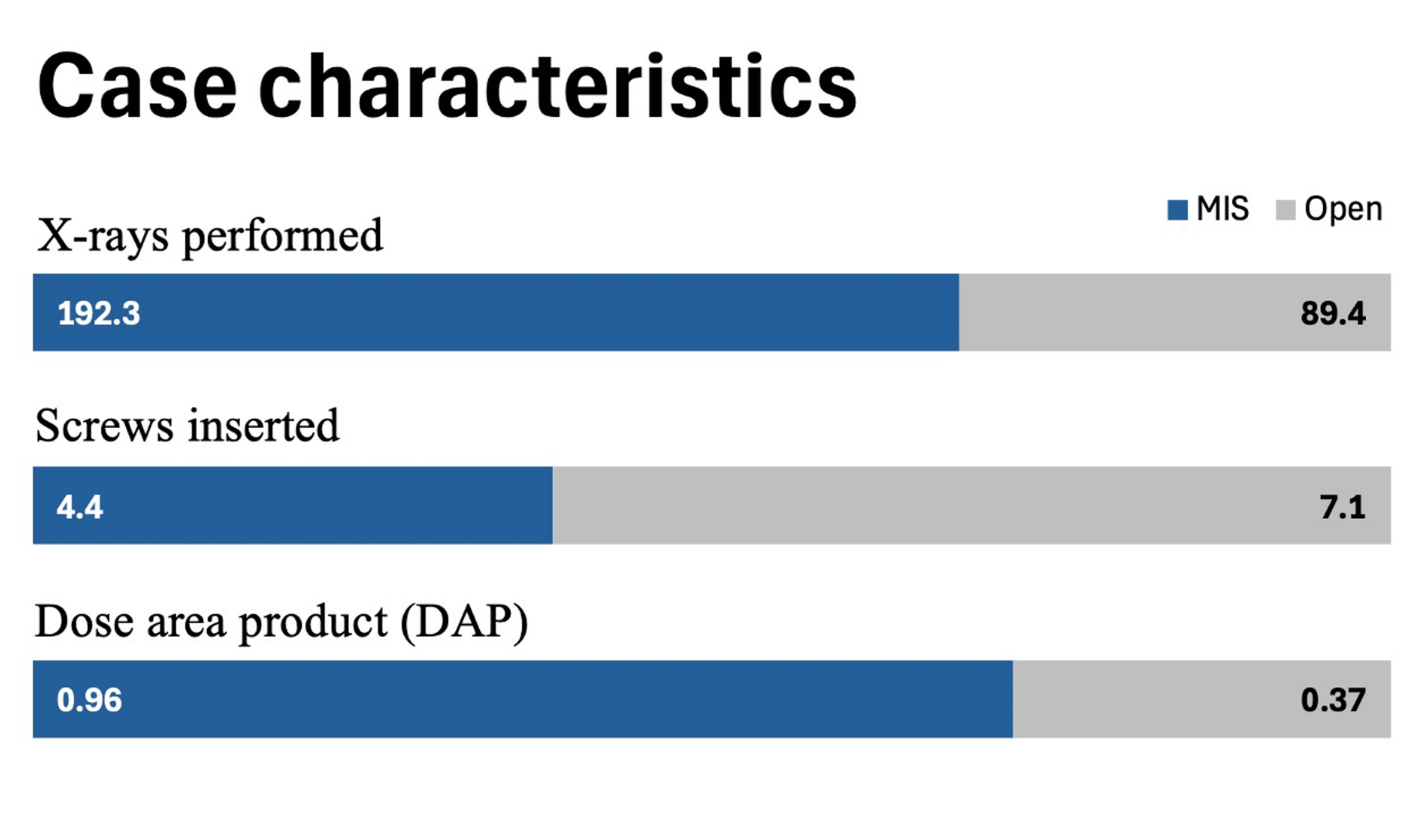
Case characteristics of minimally invasive spine (MIS) and open procedures in respect to number of X-rays performed, average number of screws inserted, and average DAP (mGy/m2)

Open procedures required significantly fewer X-rays per surgery (89.4) compared to MIS procedures (192.3) (p<0.001), resulting in lower average DAP values (0.37 mGy/m² for open vs. 0.96 mGy/m² for MIS) (p<0.001). However more screws were inserted on average in open surgery compared to MIS surgery (7.1 and 4.4 respectively) (Figure 2).

These differences underline the significantly greater radiation burden associated with MIS surgeries, largely due to the need for more fluoroscopic guidance.

The number of X-rays and corresponding DAP values increased with lower spinal levels. Cervical procedures incurred the fewest X-rays, with a gradual increase in thoracic and lumbosacral surgeries, the latter reaching an average DAP of 1.1 mGy/m² for MIS cases.

**Figure 3:**
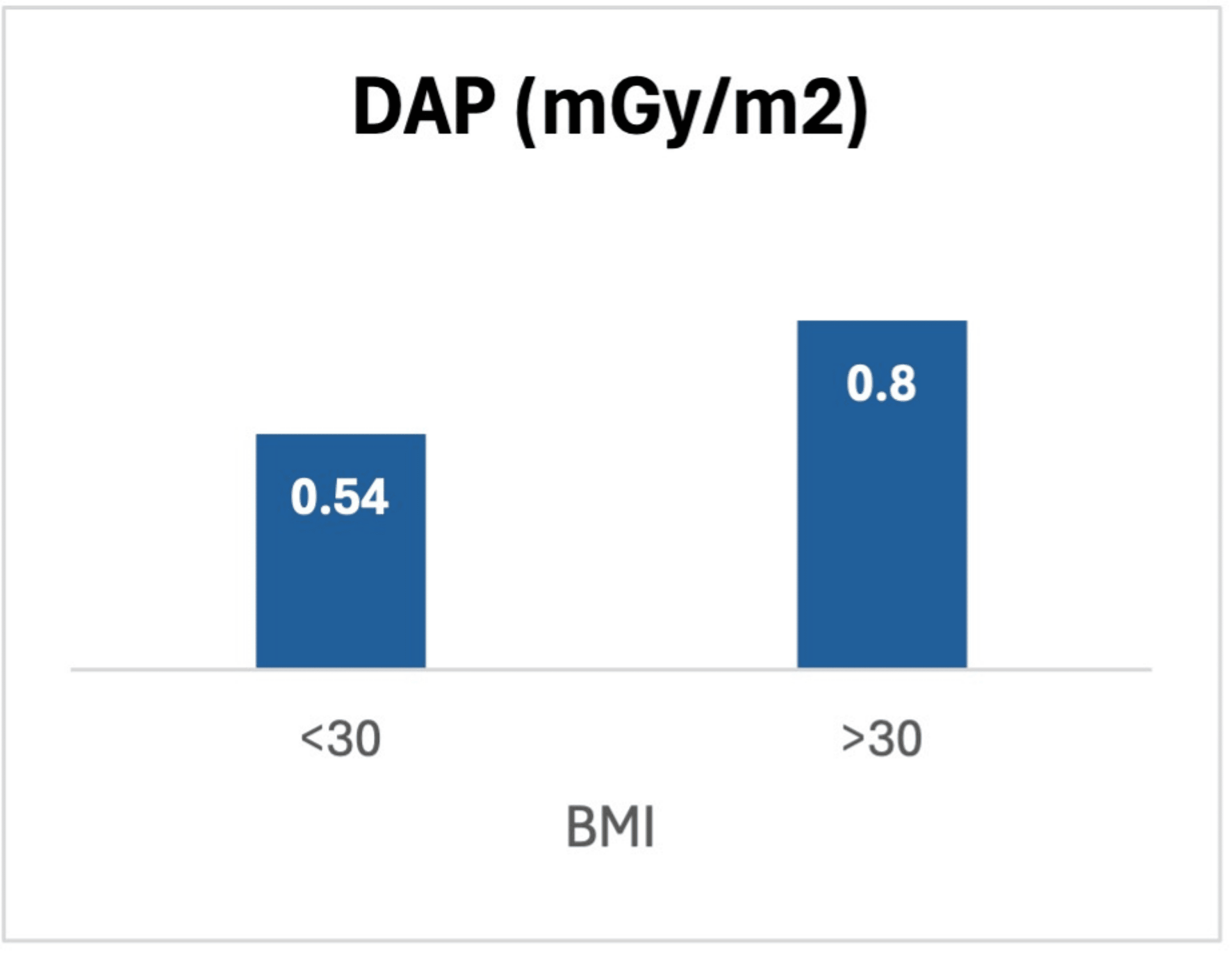
Average dose area product (DAP) (mGy/m2) exposure in patients with BMI less than and more than 30

Moreover, patient BMI was found to be a significant factor in radiation exposure. Patients with a BMI <30 (n=100) received an average DAP of 0.54 mGy/m², while those with BMI >30 (n=41) experienced a significantly higher dose of 0.8 mGy/m², despite undergoing fewer X-rays on average (p < 0.05) (Figure 3).

This suggests that higher BMI patients require greater radiation doses to achieve adequate imaging.

Finally, MIS interbody fusion procedures were associated with particularly high radiation exposure, averaging 208 X-rays per surgery and a DAP of 1.1 mGy/m². In comparison, open interbody fusions required 118 X-rays and resulted in a lower average DAP of 0.53 mGy/m² (p<0.001). These results suggest that MIS interbody fusions are among the highest radiation exposure cases.

## Discussion

Our study provides the first direct comparisons of radiation exposure in open versus MIS spinal surgery in a UK Level 1 Trauma Centre. The central finding in this study is that MIS cases required over twice the number of X-rays, leading to significantly higher radiation exposure for both the patient and the surgical team.

Other technologies are available for surgical fixation such as CT-image guided navigation and robotics. The reliance on fluoroscopy (in lack of navigation or robotics) in MIS surgery is a major factor contributing to this increased exposure [9]. Fluoroscopy, while invaluable for real-time imaging during surgery, poses inherent risks due to the need for frequent imaging to ensure the correct placement of instruments, especially in the absence of direct visualization. In open surgeries, the surgeon has a clearer anatomical view, reducing the dependency on fluoroscopy. MIS surgery, on the other hand, relies heavily on intraoperative imaging to compensate for the limited visual field and the complexity of instrument navigation through small incisions. Intra-operative imaging increases surgical accuracy in cases of X-ray-guided screw placement versus freehand but at the expense of increased radiation exposure both to surgeon and patient.

The increased radiation dose in MIS procedures can be attributed to the necessity for more precise imaging, especially when placing hardware such as pedicle screws. Fluoroscopy becomes the surrogate for direct sight, leading to prolonged exposure times and a greater number of imaging cycles. Notably, specific surgeries such as transformaminal lumbar interbody fusion (TLIF) would require additional meticulous fluoroscopy images to ensure adequate insertion of the implant. Remarkably for the operating surgeon, radiation occupational exposure limit is breached after 194 MIS TLIF surgeries [10].

**Figure 4:**
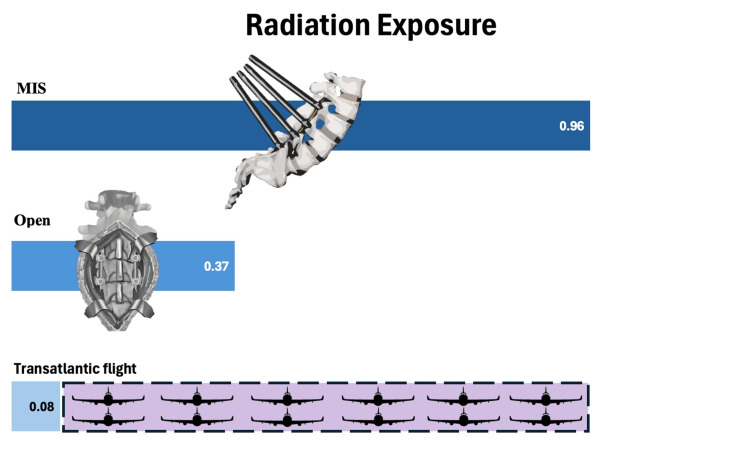
Radiation exposure for minimally invasive spine (MIS) surgery versus open surgery versus one transatlantic flight and purple shaded box showing 12 aircrafts

A transatlantic flight will expose an individual to 0.08 DAP of radiation [11]. We found in our study that patients undergoing MIS surgery have the same radiation exposure as 12 transatlantic flights during one MIS procedure (Figure 4). 

Another significant finding in this study is the impact of patient BMI on radiation dose. Interestingly the number of X-rays did not increase substantially for patients with higher BMI. The total radiation dose was significantly higher in patients with a BMI greater than 30. This is a factor not considered by many surgeons. Body habitus of the patients has a substantial impact on radiation emission [12]. For tissue penetration and to obtain the correct contrast, the X-ray machine will automatically increase the dosage. Radiation safety measures should be especially stringent for this patient group, considering the cumulative dose received during their procedures.

Given the substantial radiation exposure associated with MIS procedures and higher BMI, adherence to radiation safety protocols is critical. The principles of time, distance, and shielding remain fundamental to reducing exposure for both patients and healthcare workers. Time refers to minimizing the duration of fluoroscopic imaging, distance involves maximizing the separation between the radiation source and the surgical team, and shielding requires the use of protective barriers such as lead aprons, thyroid shields, and lead glasses. Additionally, fluoroscopy techniques such as collimation (focusing the radiation beam on a smaller area) and automatic brightness control (which adjusts the radiation dose based on image quality) are essential in minimizing radiation dose without compromising imaging clarity.

NN has emerged as a vital tool in reducing radiation exposure, especially in MIS procedures. It provides real-time 3D guidance which can dramatically reduce the need for continuous X-ray imaging by offering precise localization of instruments and implants. Studies have shown that the adoption of NN in MIS can reduce radiation exposure to the surgical team while simultaneously improving the accuracy of pedicle screw placement [13], reducing surgical time, and improving overall patient outcomes.

Despite these benefits, NN remains underutilized in many centres due to factors such as cost, availability, and the learning curve associated with its use. This study reinforces the need for broader adoption of NN technology, particularly in MIS, where the radiation risks are most pronounced. By integrating NN into standard practice, surgical teams can not only reduce radiation exposure but also increase the precision and safety of spinal procedures.

One of the limitations of this study was that we didn’t monitor direct surgeon radiation exposure or specific body parts of the surgeon to calculate specific anatomical areas that would be prone to more exposure than others. However, a systematic review found avoiding direct exposure to hands, rotation of the surgeon's body direction away from the radiation source; increasing distance from source are all effective ways to limit radiation exposure during MIS spinal procedures [14]. Another limitation was the retrospective single-centre design for this study, which we aim to avoid with future multi-centre collaborative studies on this important surgical finding. 

## Conclusions

In conclusion, this study underscores the increased radiation risks associated with fluoroscopy-guided MIS procedures, particularly in patients with high BMI. The findings advocate for the wider adoption of NN technology, which can significantly reduce radiation exposure while enhancing surgical accuracy and outcomes. As spinal surgery continues to evolve, the integration of advanced imaging and navigation tools must become a priority, particularly for MIS surgery, to mitigate the risks of radiation exposure to both patients and surgical teams. Future research should focus on identifying barriers to the widespread adoption of neuronavigation and exploring innovative imaging techniques that can further enhance the safety of spinal surgeries. This will ultimately lead to improved clinical outcomes while minimizing radiation risks, particularly in high-risk populations such as obese patients and in complex procedures like interbody fusions.
